# Supplementary material for: Lgr5 Does Not Vary Throughout the Menstrual Cycle in Endometriotic Human Eutopic Endometrium
Source: Int J Mol Sci. 2018 Dec 21;20(1):22. doi: 10.3390/ijms20010022 (PMC6337520; doi:10.3390/ijms20010022)
Supplement: Supplementary file 1 [file ijms-20-00022-s001.pdf]

International Journal of
Molecular Sciences

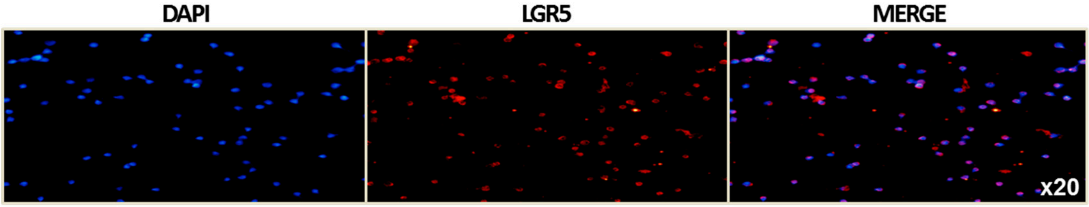

Figure S1. Cytospin LGR5. Cells sorted with NBP1-28904 antibody (for FACS) and stained with AP2745A antibody (for IF). Left panel shows cells stained by DAPI (blue); middle panel shows LGR5 in red; and right panel shows the merged images. Magnification: 20×.
